# Examining the relationship between depression knowledge level, seeking psychological help and stigma levels in patients diagnosed with depression: Preliminary results of a cross-sectional study

**DOI:** 10.1192/j.eurpsy.2025.1318

**Published:** 2025-08-26

**Authors:** I. Ersan Kiliboz, A. E. Altinöz, U. Doğan, Ö. Özer

**Affiliations:** 1 Psychiatry, Eskişehir Osmangazi University, Eskişehir; 2 Psychological Counseling and Guidance, Muğla Sıtkı Koçman University, Muğla; 3 Psychological Counselling and Guidance, Anadolu University, Eskişehir, Türkiye

## Abstract

**Introduction:**

Individuals with psychiatric illnesses still face significant stigma and discrimination around the world. These individuals are reluctant to seek treatment due to fear of stigma, and when they begin treatment, their exclusion from society is a significant obstacle to their well-being. In order for individuals with mental illness to live in a society without stigma and discrimination, it is important to increase the knowledge and understanding of that society towards mental illnesses and develop positive attitudes. Depression is quite common and is the mental disorder that causes the most disability.

**Objectives:**

This study aimed to measure the relationship between individuals’ level of knowledge about depression, seeking psychological help and stigmatization.

**Methods:**

The study group consisted of individuals who were diagnosed with major depression according to DSM 5-TR diagnostic criteria and applied to Eskişehir Osmangazi University Psychiatry Outpatient Clinic where the study would be conducted. The Hamilton Depression Rating Scale, Depression Knowledge Test, Stigma Scale, Attitudes Towards Seeking Professional Psychological Help Scale-Revised Form and Stigma Towards Seeking Psychological Help Scale were administered to the participants. Currently, 33 individuals were included in this study, and this number is planned to be 60 by the congress date. Eskişehir Osmangazi University Clinical Research Ethics Committee approved the study on 09.02.2023.

**Results:**

A total of 33 people were included in the study, 25 (75.8%) female and 8 (24.2%) male. The average age of the participants, ranging from 20 to 65 years old, was 41.48. The mean HAM-D scale score was 14.97. Education levels were calculated in years, and the mean years of education of the participants were determined to be 12.44 (5-20). According to the preliminary results of this study, a negative and significant relationship was found between depression knowledge level and stigma (rspearman: -,466, p: ,006). A negative and significant relationship was found between stigma and attitudes towards seeking psychological help (r_spearman_: -,308, p: ,029), and a positive and significant relationship was found between stigma and social stigma due to seeking psychological help (r_spearman_: -,354, p: ,043).

**Table:** 

	KTD	ASPPHS-RF	SSRPH	SS
KTD	1			
ASPPHS-RF	.206	1		
SSRPH	.016	-.331	1	
SS	-.466^**^	-.380*	.354*	1

**Conclusions:**

Stigma and stigmatization in mental illnesses are a major obstacle to diagnosis and treatment. In our study, preliminary data support the literature and show a relationship between depression knowledge level and stigma, and between stigma and psychological help-seeking behavior. These preliminary results show that psychoeducation interventions may have an important role. Results from 60 people will be presented at the congress.

**Disclosure of Interest:**

None Declared

